# Surgical Management of Sacral Chordomas: Illustrative Cases and Current Management Paradigms

**DOI:** 10.7759/cureus.301

**Published:** 2015-08-12

**Authors:** Arjun V Pendharkar, Allen L Ho, Eric S Sussman, Atman Desai

**Affiliations:** 1 Department of Neurosurgery, Stanford University School of Medicine

**Keywords:** sacral chordoma

## Abstract

Sacral chordomas represent more than 50% of all sacral tumors. These slow-growing, malignant lesions present insidiously and are often large and intimately involved with sacral neurovascular and pelvic structures. *En bloc* resection is the only well-established predictor of progression-free survival. Optimal surgical management requires a complex multi-disciplinary approach. Here, we describe two cases of sacral chordoma and review current management paradigms.

## Introduction

Chordomas are slow-growing malignant neoplasms that represent approximately 1-4% of all primary bone tumors [1]. Epidemiological studies report an incidence of 0.08 per 100,000. There is a predominance for the male gender and presentation in the 5^th^ and 6^th^ decade. Median survival is estimated to be 6.29 years. Five, 10 and 20 year survivals are approximately 68%, 40%, and 13%, respectively [2].

These tumors almost always arise in the midline axial skeleton suggesting an embryological origin from vestigial elements of the notochord [1]. Recent epidemiological studies report approximately an equal distribution between the skull base, mobile spine, and sacrum; however, chordomas represent greater than 50% of all tumors of the sacrum. Due to their relatively slow growth rate, sacral chordomas often remain clinically silent until the lesions reach a large size. When symptomatic, these tumors commonly present with non-specific and progressive deep pain and/or radiculopathy [3].

Radiological workup usually reveals a destructive bone lesion involving the vertebral body on CT, with a corresponding soft tissue mass on MRI that is T2 hyperintense and heterogeneously contrast-enhancing. There is often local invasion into the adjacent disc spaces. From a pathological perspective, chordomas are considered low to intermediate grade lesions. Due to extensive disease progression at the time of presentation and the high rate of local recurrence, these lesions are considered malignant and require multi-disciplinary management.

Here, we report two recent cases of sacral chordoma treated with *en bloc* resection and stereotactic radiosurgery and review current management paradigms for this challenging clinical entity.

## Case presentation

Informed patient consent was obtained for all participants in this report. No identifying patient information was included,

### Patient 1

**Figure 1:**
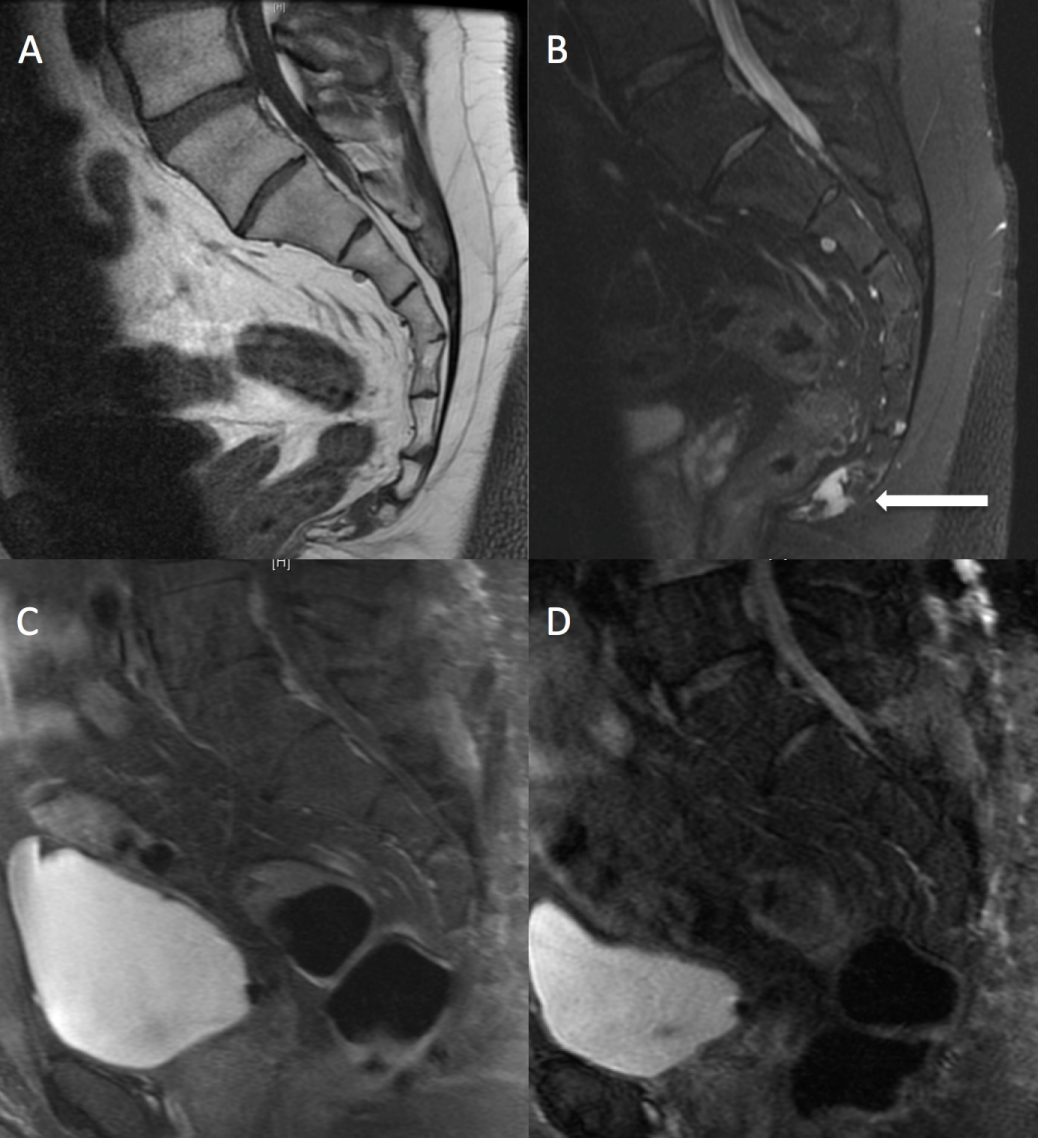
Patient 1 - Preoperative and postoperative imaging Preoperative MRI (A, B) demonstrates T2 intense well-circumscribed sacrococcygeal mass approximately 3 x 2 x 2 cm in size involving the S4 nerve roots. Postoperative MRI (C, D) shows en bloc total resection of tumor.

The patient is a 34-year-old previously healthy male who presented with three years of progressive sacrococcygeal pain, worse with sitting. He denied lower extremity weakness or sensory changes and did not have any bowel or bladder incontinence. On examination, he had no neurological deficits and had tenderness to palpation over the distal sacrum and coccyx. MRI revealed a T2 intense well-circumscribed sacrococcygeal mass approximately 3 x 2 x 2 cm in size involving the S4 nerve roots (Figure 1). The patient was taken to the operating room in conjunction with colorectal and plastic surgery specialists for a low sacral amputation and *en bloc* resection of the lesion. The patient tolerated the procedure well and was discharged home on postoperative day 1. Pathology was consistent with a chordoma with negative surgical margins. A postoperative MRI demonstrated no residual tumor. Given that there was *en bloc* resection of the tumor with negative margins, adjuvant radiotherapy was deferred. At his three-month follow-up, the patient was doing extremely well with resolved pain, a well-healed incision, and no neurological deficits nor bowel or bladder deficits.

### Patient 2

The patient is a 77-year-old previously healthy female who, after a relatively minor fall, was incidentally found to have a 4 x 2 x 4 cm sacral lesion involving the S2, S3, S4, and S5 nerve roots on CT and MRI. She underwent a CT-guided biopsy demonstrating chordoma. She was asymptomatic at the time of diagnosis and initially elected to pursue close monitoring only. Over the next six months, she began to experience progressive sacral pain. Repeat imaging demonstrated stable tumor size (Figure #2). Options of *en bloc* resection and radiosurgery were discussed with the patient. Given her advanced age and invasion of the tumor into the S2 nerve roots, the decision was made to treat the tumor with radiosurgery alone. She was treated with CyberKnife radiosurgery with a dose of 40 Gy in five sessions (Figure 3). Her follow-up is currently limited, but the patient tolerated the procedure well and was symptom-free six weeks postoperatively. 

**Figure 2:**
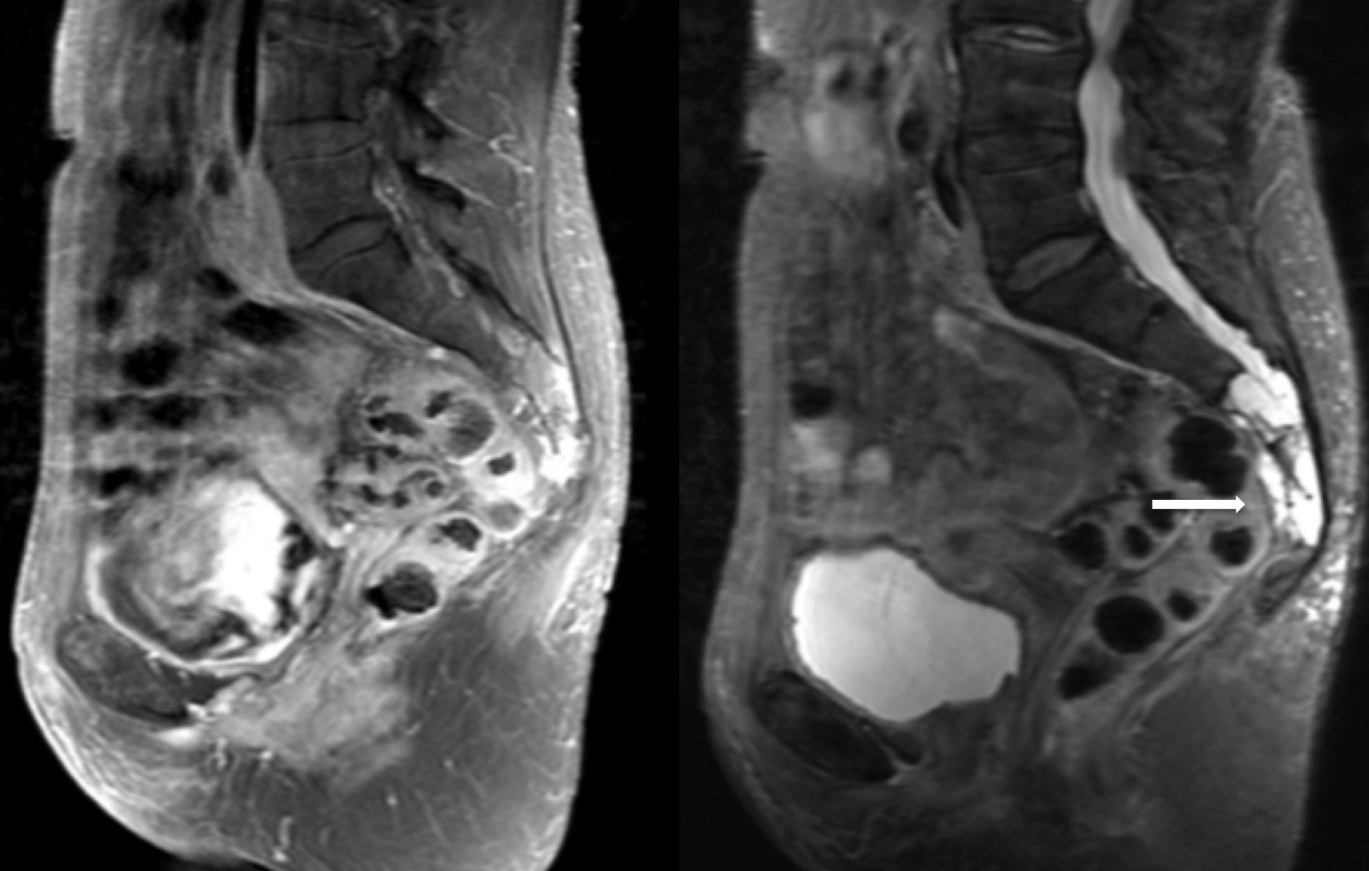
Patient 2 - Imaging findings MRI demonstrates a 4 x 2 x 4 cm sacral lesion involving the S2, S3, S4, and S5 nerve roots.

**Figure 3:**
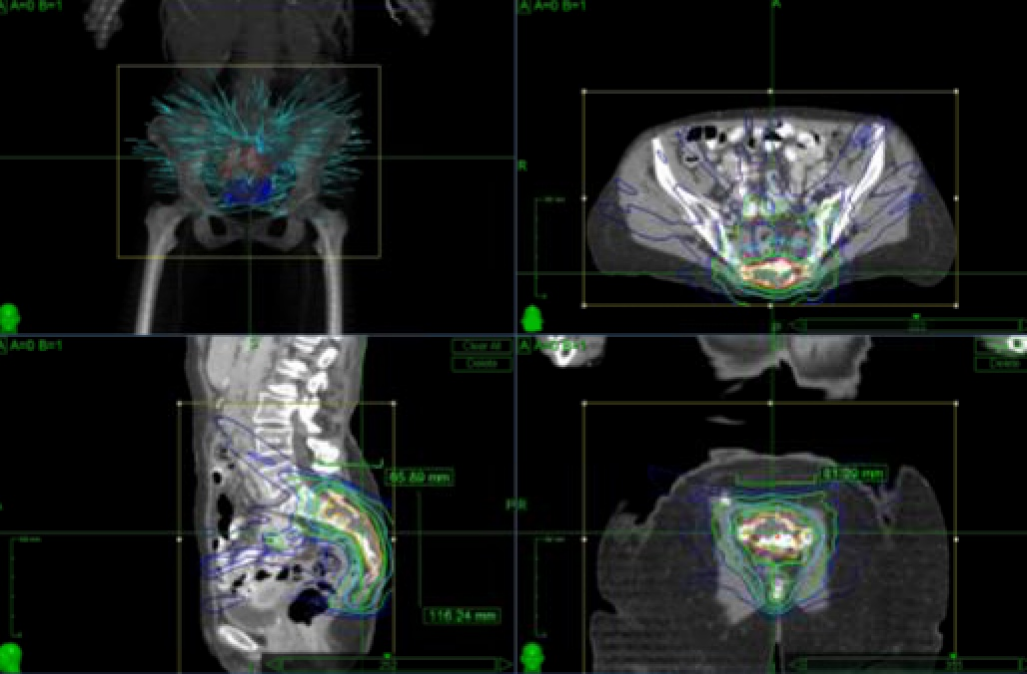
Patient 2 - CyberKnife radiosurgery plan 40 Gy was delivered in 5 sessions.

## Discussion

### Management strategies

Sacral chordomas represent a challenging clinical entity due to often large tumor size and advanced disease progression at the time of presentation. Although treatment can require multiple disciplines and incurs a risk of morbidity and decreased quality of life, surgical resection remains the definitive method of preventing local recurrence and minimizing overall mortality [4-5].

Kaiser, et al. first demonstrated the superiority of *en bloc* resection using a posterior approach in sacrococcygeal chordoma. Complete excision of the tumor without contamination of the surgical wound resulted in a 28% recurrence rate compared to 64% with subtotal resection [6]. Subsequent studies have corroborated these findings and expanded on other prognostic indicators, including tumor size > 8 cm, infiltration of the sacroiliac joints and/or adjacent musculature, and gluteus maximus or piriformis invasion [7].

### Surgical principles

The operative approach to sacral chordomas is tailored to lesion size and relationship to the sacrum, sacroiliac joints, and sacral nerve roots. Combined anterior-posterior approaches may be required in some circumstances. Lumbopelvic reconstruction with instrumented fusion is recommended in cases involving the majority of the sacroiliac joint, or when a total or high sacrectomy is performed and can be achieved using the modified Galveston technique [8-9]. For mid- and low-sacral chordomas, due to the preservation of the sacroiliac joint, lumbopelvic reconstruction is not typically required. Disconnection of the anococcygeal ligament and safe dissection of the tumor from the ventral pelvic structures is often aided by collaboration with a colorectal surgeon. Postoperative complications related to pressure-dependent wound breakdown and infection are a major source of morbidity following sacrectomy [10], and therefore, collaboration with plastic surgery for a layered wound closure with or without a flap, is beneficial.

### Neurological outcomes

With the goal of total resection, patients with sacral chordomas can experience postoperative morbidity related to motor, bowel and bladder function. The most significant predictors of postoperative function are preoperative function and level of sacrectomy [11]. The sacrifice of the S2 nerve roots and roots distal to this can risk impaired postoperative urinary and bowel function [12]. Rates for high sacrectomy are near 100% for moderate to severe postoperative bowel and bladder dysfunction but decrease to 75% and 12.5% with mid or lower sacrectomy, respectively. Reported rates of bowel/bladder dysfunction after total sacrectomy involving resection of S1 roots are also close to 100%. Resection of S1 nerve roots can also increase the incidence of postoperative plantar flexion weakness and requirement of ankle orthosis for ambulation in approximately 40% of patients [12]. Bilateral resection of sacral nerve roots involving S2-S5 results in 100% bowel and bladder dysfunction. Bilateral S2 sparing yields 40% and 25% preservation of bowel and bladder function, respectively and improves to 100% and 69% when preserving S2 and S3 roots. Unilateral nerve root sparing is associated with improved neurological outcomes and recovery with a return of function at approximately six to eight months [13]. Unilateral preservation of S3 carries a 67% and 60% chance of intact bowel/bladder function suggesting that a majority of patients can retain an adequate quality of life post-sacrectomy [14].

### Radiosurgery

Chordomas are considered to be poorly responsive to traditional radiation therapy techniques [15]. Development of stereotactic radiosurgery techniques, however, raises the possibility of increased dose application to the tumor with improved outcomes. Several papers have reported good outcomes with high dose per fraction regimens as a salvage therapy for patients who could not undergo surgical resection. Five-year local control rates are estimated between 35-60% while overall survival rate is approximately 74% [16]. High-dose single-fraction stereotactic radiosurgery has been shown to control local disease progression in up to 95% of patients at 24 months [17].

The role of adjuvant radiation therapy remains controversial. Several studies have failed to show a benefit of adjuvant therapy when *en bloc* resection is achieved [18]. One group has reported a trend towards an increase in overall survival after *en bloc *resection and initial radiotherapy [15]. There has also been a reported increase by approximately 16 months in disease-free survival with adjuvant radiotherapy after both subtotal or radical resection [4]. Proton beam therapy may also represent a promising therapeutic avenue. Five-year local control rates on patients with surgery and radiation are reported as 90% for primary and 57% for recurrent lesions [19]. Radiotherapy may also delay the time to local recurrence specifically in patients with partial resection [20-22]. 

### Reoperation after recurrence

When recurrence does occur, it commonly involves the soft tissues around the sacrum, including the piriformis and gluteus maximus muscles. There may be a role for reoperation after recurrence but only with complete resection [23]. These cases are noted to be exceptionally difficult due to scarring and obscured tumor margins.  

## Conclusions

Sacral chordoma is a complex clinical entity which often presents in a delayed fashion leading to large tumor size and involvement of critical neural elements in the sacrococcygeal region. For those patients who can tolerate the operation, *en bloc* resection with a multi-disciplinary team of colorectal and wound specialists is the gold standard for limiting recurrence and maximizing survival. The role of radiotherapy in an adjuvant role or in recurrence remains unclear. For patients who cannot tolerate an operation, radiation may provide a less optimal option for disease control in a limited fashion. 
